# Participatory Action Research Among People With Serious Mental Illness: A Scoping Review

**DOI:** 10.1177/10497323231208111

**Published:** 2023-11-06

**Authors:** Elizabethmary Thomas, Tanya Elizabeth Benjamin-Thomas, Abirame Sithambaram, Janki Shankar, Shu-Ping Chen

**Affiliations:** 1 Faculty of Rehabilitation Medicine, 70412 College of Health Sciences University of Alberta, Edmonton, AB, Canada; 2 School of Occupational Therapy, 2910 Texas Woman’s University, Houston, TX, USA; 3 Department of Occupational Therapy, Rocky Mountain University of Health Professions (Online program), Colombo, Sri Lanka; 4 Faculty of Social Work, 2129 University of Calgary, Edmonton, AB, Canada

**Keywords:** mental health, community-based participatory action research, psychosis, empowerment, patient-led research

## Abstract

Participatory action research (PAR) is a research approach that creates spaces for marginalized individuals and communities to be co-researchers to guide relevant social change. While working toward social transformation, all members of the PAR team often experience personal transformation. Engaging people with serious mental illness (PSMI) in PAR helps them to develop skills and build relationships with stakeholders in their communities. It supports positive changes that persist after the completion of the formal research project. With the increasing recognition of PAR’s value in PSMI, it is helpful to consider the challenges and advantages of this approach to research with this population. This review aimed at determining how PAR has been conducted with PSMI and at summarizing strategies used to empower PSMI as co-researchers by engaging them in research. This scoping review followed five steps Arkesy and O’Malley (2005) outlined. We charted, collated, and summarized relevant information from 87 studies that met the inclusion criteria. We identified five strategies to empower PSMI through PAR. These are to build capacity, balance power distribution, create collaborative environments, promote peer support, and enhance their engagement as co-researchers. In conclusion, PAR is an efficient research approach to engage PSMI. Further, PSMI who engage in PAR may benefit from strategies for empowerment that meet their unique needs as co-researchers.

Participatory action research (PAR) is a research approach that embodies an emancipatory agenda and seeks to work with community members as co-researchers in building knowledge and guiding change (Fals-Borda & Rahman, 1991; Reason & Bradbury, 2005). The traditional separation of knowledge generation and action in research has been identified as problematic (Jacobs, 2018). However, PAR seeks to bridge this gap by integrating both into a single continuum (Benjamin-Thomas et al., 2018). PAR has three primary goals: generating practical, locally relevant knowledge, making knowledge accessible, and promoting individual and social transformation to meet the participants’ needs (Schneider, 2012). What sets PAR apart from other research methods is its focus on equitable partnerships between researchers and communities, as well as its commitment to positive social change (Brydon-Miller et al., 2020; Wallerstein & Duran, 2017).

We can realize PAR’s full potential by embodying its central tenets and principles. These include the sharing and negotiation of power, mutual respect, and maximizing equitable participation of people with serious mental illness (PSMI) by helping them make informed decisions throughout the research process to guide relevant change (Benjamin-Thomas et al., 2018). According to Grant and colleagues (2008), managing resources, negotiating differences of opinion about the research process, and adapting to changes in the planned timeline can pose practical, philosophical, and interpersonal challenges in PAR. These challenges may arise due to participants falling ill, discontinuing participation, or requiring extra training and support. The level of co-researcher engagement and power negotiation between academic and community partners may vary at different stages within a research project depending upon such factors as level of interest, time available, experience, and resources available.

Understanding the value of PAR with PSMI, the challenges faced in carrying out PAR, and suggestions for practice from previous research (Desai et al., 2019; Schrank & Wallcraft et al., 2009) will help future researchers engage PSMI as co-researchers further. Several reviews have addressed the use of PAR with PSMI. Of two notable qualitative systematic reviews, one is on the experiences of young women with psychosis and their relationships (Chernomas et al., 2017). The other is about the complexities of sexuality and intimacy among PSMI (McCann et al., 2019). Further, there is an integrative review on consumer research to improve PSMI’s physical health outcomes (Happell & Roper, 2007) and a scoping review that explores the way that PAR can enhance young people’s mental health and resilience (Raanaas et al., 2020). However, no reviewer has mapped the nature and extent of PAR with PSMI to date. The rationale for this scoping review was derived from the need to address transformation at both the community and individual levels in a better way. To this end, we compiled insights from published PAR studies on research priorities, methods and methodologies used, strategies to empower PSMI, and ways to involve them in PAR.

The overarching goal of this review was to examine the documented use of participatory action research with PSMI. The specific objectives were to understand PSMI’s levels of engagement in the PAR process, trends in the research areas prioritized in PAR with PSMI, the various methods and methodologies used within PAR with PSMI, techniques to ensure methodological rigor, ways to facilitate personal and social changes, and strategies to enhance PSMI’s meaningful participation in various stages of PAR.

## Methods

### Study Design

A scoping review was considered an appropriate method, as it facilitates a bird’s eye view of the research available on the extent and range of the research activity. We combined the approach Arksey and O’Malley (2005) described with recommendations by Levac and colleagues (2010). The review followed five steps: (a) identify the research question; (b) search for relevant studies; (c) select studies; (d) chart the data; and (e) collate, summarize, interpret, and report the results.

#### Step 1: Identification of the Research Question

The research question that guided the review was “What is known about the use of PAR with people with serious mental illness?” We define the term PAR with PSMI as “a research approach for enabling people diagnosed with mental health problems to take part in carrying out research and in doing so to promote health equity, citizenship, and social justice for people with a mental health diagnosis” (Schneider, 2012, p. 153). Community-based PAR (CBPR) is a research approach in which researchers, community organizations, and community members, particularly people with lived experience of mental illness, engage in the research process collaboratively to effect social transformation (Sayer et al., 2019). This review uses PAR and CBPR synonymously to describe research that engages individuals and communities in collaborative knowledge generation and action. Serious mental illness (SMI) is a mental, behavioral, or emotional disorder that results in severe functional impairment and interferes with or substantially limits one or more major life activities (National Institute of Mental Health, 2023). Although SMI encompasses a variety of diagnoses, we use the term PSMI in this study to describe individuals with a diagnosis of schizophrenia, bipolar disorder, and any form of a psychotic disorder that severely marginalizes people living with SMI (National Collaborating Centre for Mental Health, 2014).

#### Step 2: Identification of Relevant Studies

Six electronic databases were searched in January 2022 using the relevant keywords and appropriate searching syntaxes. Of these, five major social science databases, including Medline, Embase, APA PsycINFO, CINAHL, and Scopus, were chosen to identify articles from various disciplines. We included grey literature (ProQuest Dissertations and Theses) in our search to ensure a comprehensive overview of the research topic (Levac et al., 2010). The main concepts “participatory action research” and “people with serious mental illness” were each cross-referenced using relevant keywords, including “participatory action research,” “community-based participatory research,” and “Photovoice” for PAR, and “schizophrenia,” “bipolar,” and “psychotic disorder” for PSMI. No methodological or time limits were employed. We sorted the duplicates manually and scanned critical articles’ reference lists to ensure a thorough search.

#### Step 3: Selection of Studies

Two independent researchers (ET and AS) screened the titles and abstracts, while ET screened the full text after that to identify studies that met the inclusion criteria. These criteria included studies published in English that used PAR or CBPR with PSMI and engaged them in the research process. During the full-text screening, we used the Critical Review Form—Qualitative Studies, v. 2.0 (Letts et al., 2007) to appraise articles concerning the criteria, including study design, descriptive clarity, and procedural rigor. We excluded articles that studied individuals with diagnoses other than those specified in our definition of PSMI.

**Figure 1.**
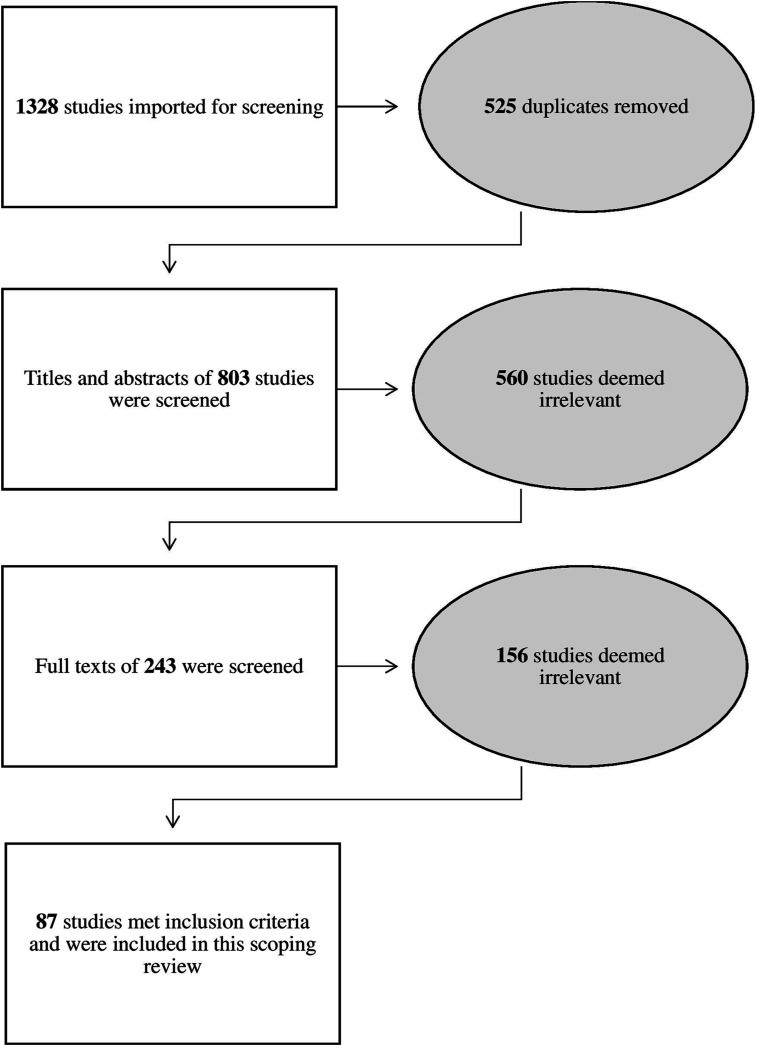
PRISMA flow chart.

The PRISMA flowchart (Figure 1) shows that the search yielded 1328 results. We identified and removed 525 duplicates and screened the remaining 803 titles and abstracts for their relevance to the inclusion criteria, and 560 studies were deemed irrelevant and excluded at this stage. Of the remaining 232 studies, we excluded 145 because they did not meet the inclusion criteria. This review included the remaining 87 studies, of which 10% (nine dissertations) were from grey literature.

#### Step 4: Charting of the Data

We used a descriptive-analytical method (Arksey & O’Malley, 2005) that applied a common analytical framework to the 87 studies included. We charted the general citation information, the study population’s demographic characteristics, the study’s purpose, methods used, key findings, the level of engagement, and strategies used to empower PSMI during the research process using the Covidence software (Covidence, 2022). Then, we exported the data as an Excel file for further analysis.

#### Step 5: Collation, Summarization, Interpretation, and Reporting of Results

We created a descriptive summary of the findings using a rudimentary descriptive analysis of the extent, nature, and distribution of the studies included. Directed content analysis, as Hsieh and Shannon (2005) described, was used to analyze and present the areas of research focus, the PSMI’s level of engagement in the research process, the specific strategies used for individual and social transformation with PSMI, the challenges faced during the research, and the measures used to ensure the trustworthiness of the studies included.

## Results

### General Description of the Studies Included

#### Demographic Information

Table 1 provides a complete list of the 87 studies included—the years of publication range from 1997 to 2021. Although a large proportion of the studies (70%) recruited participants who had been diagnosed with psychotic illnesses, 14 (16%) and 13 (14%) of the studies focused specifically on people who experience bipolar disorder and schizophrenia, respectively. Of the 87 studies, more than two-thirds (74.7%) were published in North America, 17% in Europe, and 3% in Australia. One study was international, and one study each was from Singapore (P55), China (P56), and India (P65).

**Table 1.** Areas of Research Focus and Complete List of the Included Articles (*N* = 87).

Research focus	Number of studies (*n*)	List of included articles
*Activity and participation areas: 29*		
Self-care	12	P1 (Gorczynski et al., 2013); P2 (Michalak et al., 2019); P3 (Sims-Gould et al., 2017); P4 (Lapadat et al., 2020); P5 (Kahn et al., 2012); P6 (Weinstein et al., 2019); P7 (Morton et al., 2022); P8 (Stanhope & Henwood, 2014); P9 (Michalak et al., 2016); P10 (Weinstein, 2018); P11 (Sayer et al., 2019); P12 (Sameby et al., 2008)
Employment and leisure	6	P13 (Rebeiro, 2012); P14 (Rebeiro Gruhl et al., 2012); P15 (Gruhl et al., 2012); P16 (Sundar & Ochocka, 2004); P17 (Stone et al., 2018); P18 (Johnson et al., 2016)
Social participation	11	P19 (Dulek et al., 2021); P20 (Skoy & Werremeyer, 2019); P21 (Suto et al., 2021); P22 (Rowe et al., 2012); P23 (Quintas & Burnett, 2013); P24 (Barrow et al., 2014); P25 (Terp et al., 2016); P26 (Onken, 2018); P27 (Millner et al., 2019); P28 (Agner et al., 2020); P29 (Petros et al., 2021)
*Environmental aspects: 41*		
Support and relationships	2	P30 (Timmers et al., 2014); P31 (Schneider et al., 2004)
Stigma	5	P32 (Suto et al., 2012); P33 (Russinova et al., 2018); P34 (Thai et al., 2021); P35 (Roelandt et al., 2020); P36 (Desai et al., 2019)
*Services, systems, and policies: 34*		
Clients’ experiences of using mental health services	21	P37 (Davidson, 1997); P38 (Rose et al., 2008); P39 (Morgan et al., 2003); P40 (Magliano et al., 2009); P41 (Barbato et al., 2014); P42 (Larkin et al., 2015); P43 (Slater et al., 2016); P44 (P. W. Corrigan et al., 2017); P45 (Iezzoni et al., 2018); P46 (Du Bois et al., 2020); P47 (Sylvestre et al., 2007); P48 (Tischler et al., 2010); P49 (Pelletier et al., 2015); P50 (Makdisi et al., 2013); P51 (P. Corrigan et al., 2015); P52 (Lindamer et al., 2008); P53 (Michalak et al., 2012); P54 (Fernandes, R.M., 2012); P55 (Maniam et al., 2016); P56 (Chan et al., 2019); P57 (Olausson et al., 2021)
Housing	5	P58 (Montgomery et al., 2008); P59 (Padgett et al., 2013); P60 (Thompson et al., 2008); P61 (Cabassa et al., 2013); P62 (Bonugli, 2014)
Feasibility of novel interventions	8	P63 (Guy et al., 2020); P64 (Malling, 2013); P65 (Ramaswamy, 2014); P66 (Whitley et al., 2020); P67 (Weinstein et al., 2021); P68 (Karadzhov, 2021); P69 (Sikstrom et al., 2020); P70 (Abram et al., 2020)
*Overall recovery conception: 17*		
Recovery	15	P71 (Robertson et al., 2020); P72 (Raymaker et al., 2020); P73 (Stoffel, 2007); P74 (Ochocka et al., 2005); P75 (Michalak et al., 2012); P76 (Cabassa et al., 2013); P77 (Mizock et al., 2014); P78 (Mizock et al., 2015); P79 (Solomon et al., 2016); P80 (Thompson, 2015); P81 (Rouse et al., 2017); P82 (Yung, 2018); P83 (Toney et al., 2018); P84 (Vansteenkiste et al., 2021); P85 (Paton et al., 2018)
Resilience	2	P86 (Lal, 2012); P87 (Deegan, P. E., 2005)

#### Level of Co-researchers Engagement

Research has shown the value of developing partnerships with the community to facilitate PSMI’s meaningful participation (Rebeiro Gruhl, 2012). One of PAR’s main principles is to involve participants as co-researchers actively within various phases of the research process (Baum et al., 2006; Hacker, 2013). The studies that used a CBPR approach reported the value of long-standing partnerships with community-based organizations, such as supportive housing agencies (P6) and local mental health agencies (P27), to create space for PSMI co-researchers to determine the research priorities, choice of methods, and use of the knowledge generated. This approach is exemplified in peer-directed research by the collaborative research team that studies psychosocial issues in bipolar disorder (CREST BD). This Canadian research network focuses on applying CBPR in bipolar disorder research (P2, 18, 32, and 75).

Similarly, 12 studies that described a PAR approach have engaged PSMI in all stages of the research process (P17, 23, 31, 40, 47, 50, 64, 66, 70, 71, 84, and 85). PSMI co-researchers were part of an advisory group with relevant stakeholders, which allowed them to guide the research priorities and the methods used and take ownership of the knowledge generated. Examples of advisory groups include a steering committee (P16) to improve workplace mental health and a “PAR co-design team” to explore recovery from psychosis and resilience (P34).

The articles exhibited variations in how PSMI set research priorities, determined data generation methods, and disseminated the findings. Eight studies (P28, 33, 40, 41, 50, 61, 63, and 66) elaborated on the role that PSMI play in determining the research priorities (e.g., facilitators and barriers to living with psychosis by a group of students with lived experience, P50). Six studies described the way PSMI co-researchers engaged in generating knowledge by conducting in-depth interviews (P74), focus group discussions (P31), developing workshops (P34), and videos that describe ways to reduce mental health stigma (P66). Eight authors reported engaging co-researchers in data analysis through methods such as consumer concept mapping (P26), developing narratives for their photographs in a Photovoice study (P73), and engaging in group discussions (P37, 50, 64, 70, 74, and 84).

Participant-led ways of sharing the knowledge acquired included community forums (P14), a group theatre presentation (P31), a meet the authors event (P36), community displays of artwork (P80), screening videos to reduce mental health stigma (P66), and distribution of a list of strategies to improve housing facilities to policymakers (P47). In most Photovoice studies, co-researchers hosted a public display of their photos to encourage conversations about their perspectives (e.g., P1, 55, and 84). Other methods of sharing knowledge reported include peer-led websites (P9) and including peers as co-authors in publications (e.g., P31, 71, and 87). Several papers referred to study participants as “service user researchers” and “participant co-researchers” (e.g., P6, 71, and 82).

#### Research Areas Prioritized in the Studies

**Figure 2.**
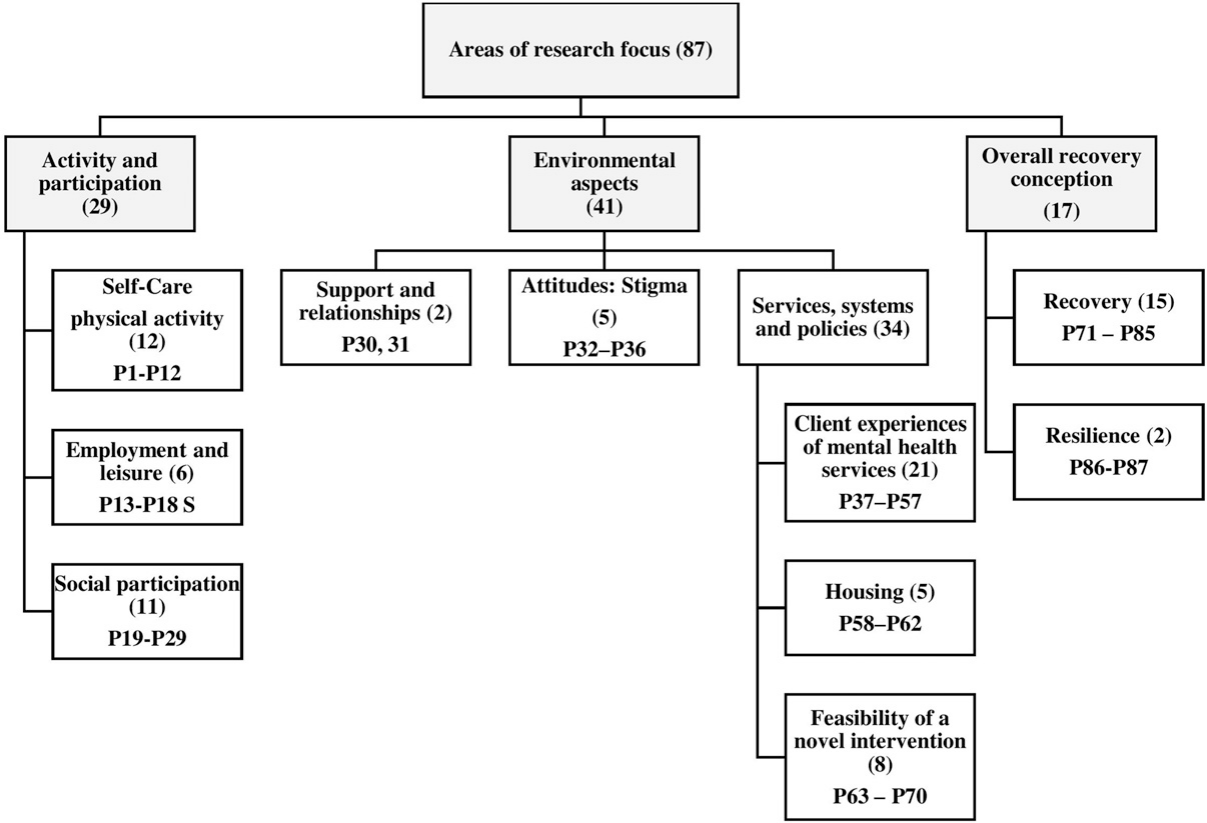
Areas of research focus (*N* = 87).

The studies in this review prioritized three main research areas: activity and participation, environmental aspects, and PSMI’s overall conception of recovery (see Table 1 and Figure 2). We used the ICF (World Health Organization, 2001) as a guiding framework to identify critical sub-categories. We found that 29 of the 87 articles (33%) focused on activity and participation and included such topics as self-care, interpersonal interactions, and responsibilities, such as fulfilling the role of parenting (P24), access to, and engagement in, significant life areas like employment (P17), and social participation. As shown in Table 1, 12 studies explored participant-oriented interventions to promote self-care, such as increased physical activity (P3), healthy food choices (P10), and digital self-management strategies (P2).

Approximately half of the studies included (*n* = 41, 47%) explored environmental aspects that affected PSMI, including topics such as the presence of stigma (P33), challenges in supportive housing (P58), and client-led evaluation of mental health services (P38). For example, 24 studies reported topics related to clients’ understanding of schizophrenia’s causes and consequences (P40) and developed a questionnaire to assess the quality of mental healthcare (P41). The participants’ conception of recovery was the focus of 20% (*n* = 17) of the studies. It included topics such as the personal meaning of recovery (P75), the effect of the participatory clubhouse model on the recovery process (P73), and the development of resilience (P86).

#### Methods Used in the Studies

PAR allows for flexibility concerning methods and methodologies used to generate and disseminate data. The studies included in this review reported using a PAR or CBPR approach with various qualitative and mixed-method designs that suited their research question. Most (*n* = 68, 78%) used qualitative methods, among which 45 studies (52%) used focus group discussions (FGDs) and 21 (24%) used the Photovoice methodology. Other qualitative methods used included in-depth interviews (e.g., P1, 21, and 68), group discussions (P5), concept mapping (P26), and participatory video (P66). A mixed-methods design was employed by 19 (22%) studies, with such quantitative methods as pre- and post-intervention surveys (e.g., P29, 38, and 78) and cluster analysis (P46) in conjunction with qualitative methods such as FGDs.

#### Methodological Rigor of the Studies Included

Methodological rigor is essential in qualitative research and applies to participatory research methods (Tracy, 2010). Most of the studies reviewed reported using methods that Lincoln and Guba (1985, Yardley (2017), and Padgett (2016) recommended to ensure trustworthiness throughout the research process. In this review, 34 authors (39%) utilized member checking to verify that their preliminary findings accurately reflected the collective experience of their co-researchers (e.g., P6, 31, and 65). Additionally, 17 (19.5%) authors described triangulation (e.g., P11, 49, and 56) and combined methods such as in-depth interviews and focus group discussions with Photovoice to develop a more comprehensive understanding of their findings. Six (6.9%) studies reported engaging PSMI in data analysis (P6, 10, 31, 57, 64, and 86) to ensure credibility. For example, a study of healthy living among PSMI enlisted interested co-researchers to read anonymized transcripts of focus group discussions to identify preliminary themes (Weinstein et al., 2019). A majority (81, 93%) of the studies used purposeful sampling, and 25 (28.7%) provided detailed descriptions of the co-researchers and research site, including verbatim quotes by co-researchers to ensure a detailed description of the context, and allowed the reader to determine the way that the findings applied to their contexts (e.g., P13, 22, and 65). However, very few studies provided reflexive notes on the researchers’ positionality, theoretical perspectives, and biases or the challenges faced within the research process (e.g., P31, 64, and 82).

#### Individual and Social Change

Collaborative action to effect positive change is a crucial tenet of PAR (Benjamin-Thomas et al., 2018). The research process and its outcomes foster such change on multiple levels, including personal, local, regional, and national (Brydon-Miller et al., 2020). PSMI gained new skills, including research skills, coping strategies for stigma (P33), new knowledge concerning self-management techniques and healthy food options (e.g., P3, 25, and 50), increased self-awareness (P59), and reduced self-stigma (P66). They described empowerment as having a seat at the table (P34), being listened to (P48), and being able to use their voice to influence policy change (P58).

Social change was evident in the increased local awareness of PSMI’s concerns, such as difficulty in accessing healthy food options (P1), lived experience of homelessness (P59), and challenges in supportive housing (P47), which led to a deeper understanding of mental illness stigma’s effect (P33, 50, and 60). One study on college students with mental illness resulted in a campus-wide campaign to encourage discussion about mental health (P20). Collaboration with community-based organizations and peer-led groups in the research process facilitated the development of supportive social networks, such as a community garden (P21) and physical activity groups (P3), some of which continued after the formal research process (e.g., P4, 28, and 61). The action taken by research groups influenced policy change concerning employment opportunities (P13) and supportive housing facilities (P58).

### Strategies to Empower PSMI Through Engagement in PAR

**Figure 3.**
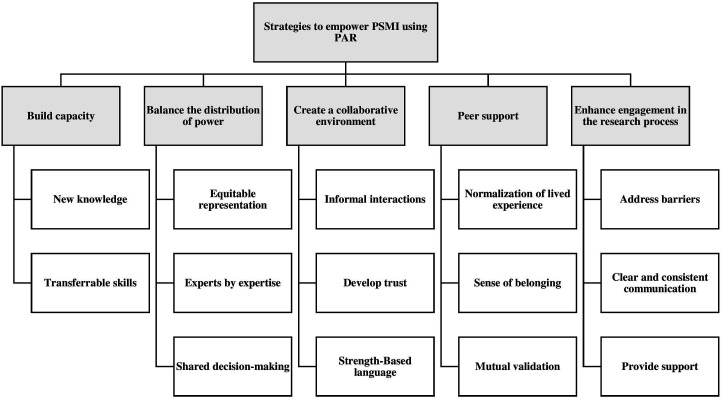
Strategies to empower PSMI through engagement in PAR.

Sundar and Ochocka (2004) described empowerment as an opportunity for people to gain control to influence recommendations and actions needed to realize individual and social transformation. An essential goal of participatory research is to empower co-researchers to become aware of and develop agency to advocate for their own needs and those of their community (Brydon-Miller et al., 2020). We adapted Chen and Krupa’s (2018) a framework of empowerment to categorize five critical strategies used in the studies reviewed to engage and empower PSMI through PAR. These are to build capacity, balance power distribution, create a collaborative environment, promote peer support, and enhance engagement in the research process (see Figure 3).

#### Building Capacity

Engaging PSMI in research requires valuing their lived experience and creating opportunities to acquire new knowledge and skills that are meaningful to them and relevant to the research process. For example, a peer-led study (P2) developed a web-based resource called “The Living Library,” where PSMI could share experiences with a peer expert who could understand and relate to them. In a study designed to enhance young PSMI’s awareness of self-management strategies (P4), the author highlighted the importance of allowing participants longer timelines to build capacity and confidence as they gradually assumed new responsibilities. Engagement in research also facilitated the development of research skills, such as interviewing (e.g., P3, 11, and 72), research ethics (P4), data collection and analysis methods (e.g., P17, 38, and 48), and transferable skills, such as leadership (e.g., P20, 46, and 82) and group facilitation (e.g., P11, 31, and 63). These skills equipped PSMI co-researchers to think critically, recognize their role, take responsibility, and make informed decisions in various stages of the research process.

#### Balancing the Distribution of Power

The equitable distribution of power is one of PAR’s critical principles. Because previous negative research experiences took PSMI for subjects and treated researchers as experts, the research team is inherently in a powerful position compared to PSMI co-researchers (P31). Some researchers recognized the need to shift the power distribution in favor of PSMI co-researchers intentionally and used strategies such as ensuring adequate representation of PSMI co-researchers in the research team (P7, 9, and 26), valuing them as experts according to expertise (P34), acting on their recommendations (e.g., P26, 37, and 72), and providing opportunities for them to build capacity, assume responsibilities, and guide decisions throughout the research process (e.g., P38, 51, and 62). Transparency concerning the research process (P64) and an informal environment (e.g., P28, 31, and 37) helped the co-researchers share their opinions from a position of agency. Hiring PSMI as paid co-researchers (P49) and encouraging them to take ownership of their work and co-author publications (P34, 71, and 87) was also reported to promote equitable power distribution.

#### Creating a Collaborative Environment

A collaborative environment is one in which all participants are valued and can contribute to various aspects of the research process. Researchers recognized the importance of creating an affirming environment to enable PSMI co-researchers to negotiate and share power from a position of agency (P72). Studies that invested adequate time to develop long-standing partnerships with community and peer-led groups reported that familiarity with the research team and trust built over time enhanced the engagement of PSMI co-researchers in the research process (e.g., P4, 31, and 82). Strategies used to create a collaborative atmosphere included encouraging and modeling the use of strength-based language (e.g., P6, 20, and 31), inviting them to express their ideas and feelings (P64), and promoting understanding, solidarity, and socially responsible ways of relating to each other (P23). For example, in a study that explored recovery from and resilience in psychosis, the PAR Co-Design Team, including PSMI co-researchers, engaged in collaborative discussions about addressing their concerns proactively concerning ethical issues and decisions about self-identifying as people with lived experience (P34).

#### Peer Support

Many studies have reported that sharing personal challenges in a safe space promoted a feeling of normalization of individual experiences (P8, 21, and 55) and led to a sense of belonging through mutual support and affirmation (P28). In a Photovoice study on the effect of engaging in a clubhouse, PSMI described that the confidence built in Clubhouse activities helped them feel more able to pursue opportunities outside the Clubhouse (P28). Further, collaboration to achieve a common goal fostered a connection among those involved (P4). For instance, in a community gardening project, the author reported that PSMI co-researchers assumed and fulfilled roles voluntarily, which contributed to an atmosphere of belonging and strengthened their sense of purpose and place within the group further through validation from others (P21).

#### Enhancing Meaningful Participation

PSMI participants require support to meaningfully engage with the research process and experience individual and social transformation. The studies reviewed showed three critical strategies used to bolster their participation: (a) addressing barriers to participation; (b) clear and consistent communication; and (c) provision of support throughout the research process.

##### Addressing Barriers to Participation

Research has established that PSMI can contribute meaningfully and play leadership roles in the PAR process. To better support their engagement in PAR, it is crucial to understand and address neurocognitive, emotional, and practical barriers to participation that some PSMI may face. Because of their diagnosis, some may have neurocognitive difficulty concentrating, remembering information, and articulating their thoughts (P31). To overcome these challenges, researchers have used strategies to enhance engagement, such as visual aids (P42), printed information sheets (P19), reminder emails about upcoming meetings (P4), and created a safe space for PSMI to articulate and express their thoughts at their own pace (P31, 25, and 42).

Other articles described how PSMI coped with emotional challenges such as self-stigma and fears concerning the nature of their involvement in research (P23, 31, and 42). To address these barriers, researchers reported the benefits of prolonged engagement with the research team in developing trust and enhanced co-researcher engagement over time (P31). Side effects of psychotropic medications, such as fatigue and altered sleep patterns, may affect PSMI’s participation as well. Researchers have addressed this by planning research activities with PSMI collaboratively at a suitable time, reducing participants’ burden by giving them control over the nature of their engagement (P68), and creating opportunities for them to surmount these barriers and contribute significantly to various stages in the PAR process (P23, 40, and 42).

PSMI co-researchers experienced marginalization and reported challenges, including difficulty finding employment, homelessness, and being more vulnerable to comorbidities (P52). Most of the studies included reported that they provided the PSMI co-researchers with incentives, such as gift cards (P13), healthy refreshments (P29, 50, 76), and reimbursement for transportation expenses (P44), to overcome these practical barriers and encourage continued participation. For example, a study designed to improve PSMI’s access to primary care hired people with lived experience as paid co-researchers to promote equitable participation (P49). As described in the strategies above, researchers have tailored their approach to supporting PSMI to overcome barriers to participation in PAR.

##### Clear and Consistent Communication

Many researchers reported the effectiveness of using clear language and communicating with PSMI co-researchers regularly throughout the research process (P4, 39, and 69). Young adult co-researchers appreciated setting timelines collaboratively, which allowed them to balance education and work commitments with research commitments (P22). Older co-researchers in a Photovoice study appreciated written instructions and weekly calls to remind them of the theme and the instructions before the next meeting (P61). Communication about the research goals, outcomes, roles, and anticipated challenges in the research process helped co-researchers gain clarity and feel confident in participating in the research project (P42). Further, PSMI co-researchers valued opportunities to express their additional thoughts, feelings, and ideas at the end of the research project (P19, 65, and 82).

##### Providing Support Throughout the Research Process

It was imperative to intentionally provide PSMI co-researchers with ongoing support to empower them to engage fully in the research process. This support ensured that professional resources were available to help them cope with the discomfort that may arise during the group discussions (P20 and 61). To help service user participants feel supported and to help them retain a sense of control concerning the information they chose to share, their consent was sought before each step of the study (P4). They also had the option of bringing along a carer or having a quiet place to retreat (P13 and 62). Particularly during interviews that discussed sensitive issues with the participants, researchers found it useful to intentionally spend time with participants throughout the research, and they also made follow-up calls to offer support and help participants whenever necessary (P85).

## Discussion

This scoping review provided an overview of how PSMI engaged in PAR processes. Our findings included demographic information identifying the country where the research was conducted, diagnosis of PSMI co-researchers, research priorities, methods and methodologies used, and their experiences of individual and social transformation through participation in PAR. We categorized specific strategies used to empower PSMI co-researchers, including building capacity, balancing power distribution, creating a collaborative environment, promoting peer support, and enhancing meaningful participation in the research process.

Equitable participation and social transformation are central tenets of PAR (Benjamin-Thomas et al., 2018). Many articles in this review described how they encouraged the participation of PSMI co-researchers in various phases of research. However, there was significant variation in the extent of involvement of co-researchers among the studies. Some described the engagement of co-researchers as *full participation* right from the design of the study to the dissemination of information (e.g., Paton et al., 2018; Schneider et al., 2004; Whitley et al., 2020), while others used the same terms to describe engagement in generating knowledge, without participating in setting research priorities or enacting social transformation through the research process (e.g., Morgan et al., 2003; Sameby et al., 2008; Skoy & Werremeyer, 2019). Similar inconsistencies regarding how principles of *participation* and *action* are enacted within PAR have been discussed and problematized in a critical interpretative synthesis of PAR with older adults (Benjamin-Thomas et al., 2018).

PAR is particularly relevant for PSMI, as they have been traditionally excluded from influencing clinical and research decisions concerning them. The prevalent description of PSMI as *patients* or *passive objects of study* causes them to feel uncomfortable in their roles and widens the gap between them and the research team (Rempfer & Knott, 2002). To achieve PAR’s emancipatory agenda, it is crucial to bridge this gap by creating opportunities for PSMI to acquire knowledge and build the capacity to shape research priorities from a position of agency (Lapadat et al., 2020).

Within the scope of this review, the researchers organized workshops to train interested co-researchers in relevant research methods and methodologies (e.g., Russinova et al., 2018; Terp et al., 2016; Thai et al., 2021). Further, it was common for the research team members who were open to further changes on the part of PSMI co-researchers during the PAR process to set the initial research priorities (e.g., Sims-Gould et al., 2017; Weinstein et al., 2019). Benjamin-Thomas and colleagues (2018) also observed a similar trend in their critical interpretative synthesis of PAR with older adults as co-researchers. They described the importance of researchers’ pre-establishing broad, open-ended research priorities and allowing co-researchers to shape the research process. Most of the studies included long-standing partnerships with community-based organizations or with peer-led groups that catered to PSMI’s needs and facilitated the process (Quintas & Burnett, 2013; Stanhope & Henwood, 2014; Stone et al., 2018). Our findings were consistent with existing research that found that such prolonged engagement helps build trust on the PSMI participants’ part and creates space for them to shape the research priorities and methods and take ownership of the knowledge generated (Moro et al., 2022).

Reflexive accounts of research processes are significant in all forms of qualitative research. In Tracy’s (2010) eight “Big Tent” criteria for qualitative research, she emphasized the importance of practicing “honesty and transparency about the researcher’s biases, goals, and foibles as well as about how these played a role in the methods, joys, and mistakes of the research” (p. 841). Engaging in reflexivity is particularly important within PAR, as the researchers must address sharing and negotiating power continually and reflexively throughout the research process to “explore, navigate, challenge, and share the process of attempting to break traditional power differences between researcher and participants” (Benjamin-Thomas et al., 2018, pp. 10–11). All of the studies reviewed recounted information about the researchers’ professional background, the roles of those involved, and their rationale for various decisions in the research process. Several authors went beyond the basic introductory statements to offer reflexive and nuanced accounts highlighting their position as researchers, their biases, their challenges, and the rewards of carrying out PAR with PSMI.

Strategies that some authors in the studies reviewed used to describe their positionality included disclosure of their ethnicity (Du Bois et al., 2020), identity as a psychiatrist who is a mental health service user (Tischler et al., 2010), and the way that this positionality as an insider influenced their engagement, and that of their co-researchers within the research process. Some authors used “I/We statements” to describe their reflexive experiences during the research process. For example, one service user researcher shared, “At times, I had difficulty allowing the co-researchers time and space to explore what they were being asked to do … and the need at times to be pragmatic” (Robertson et al., 2020, p. 489). Novice researchers who seek to use PAR would benefit from such transparent accounts of self-reflexivity and disclosure about how seasoned researchers facilitated co-researcher engagement, coped with the challenges and unexpected difficulties they faced, and how research priorities evolved (Tracy, 2010).

While acknowledging the value of the researcher’s positionality in enhancing PSMI’s engagement in research, it is essential to note that disclosing the authors’ identities, particularly that of consumer researchers, is a delicate topic that merits careful consideration. In an article on the benefits and disadvantages of self-identification in social research, Massoud (2022) discussed how self-identification may affect researchers who belong to marginalized communities disproportionally by opening themselves and their work to devaluation. It might make researchers hesitant to disclose positionality statements. Massoud further recommended that all social researchers acknowledge their privileges and vulnerabilities regularly, as it may render positionality an integral part of social research. A judicious description of how authors shared and negotiated power differences and achieved meaningful engagement with PSMI co-researchers would help strengthen the quality of PAR with this population.

PAR calls for collaborative action to address contextual forces that shape injustices at the individual and collective levels (Benjamin-Thomas et al., 2018). It can range from raising the affected community members’ awareness of a shared concern to influencing changes in local, regional, and national practices and policies (Brydon-Miller et al., 2020). This range is evident in how studies in this review achieved *action* and *transformation*. While most described the effect of collaborative action with PSMI, 11 studies addressed the way that they achieved *transformation* through the PAR process explicitly (Barrow et al., 2014; Guy et al., 2020; Maniam et al., 2016; Robertson et al., 2020; Sayer et al., 2019; Schneider et al., 2004; Stanhope & Henwood, 2014; Sundar & Ochocka, 2004; Toney et al., 2018; Weinstein, 2018; Yung, 2018). For example, in a PAR project designed to enhance communication between clinicians and PSMI, Schneider and colleagues (2004) described social transformation and stated that PAR “empowered one small group of very marginalized people with schizophrenia to speak directly to psychiatrists and other mental health professionals about their treatment experiences and through this is contributing to change in how others with mental illnesses are treated by their healthcare professionals” (p. 576).

In future accounts of PAR, explicit descriptions of how PSMI achieved transformation in various contexts can promote its further use with this marginalized population. In addition, the need to report strategies transparently to minimize risks and protect participants, communicate clearly about data ownership, and explore ways to educate ethical review boards to ensure that research models are consistent with PAR’s principles must be addressed continually in scholarship (Campbell-Page & Shaw-Ridley, 2013). Further, PAR researchers need to advocate for increasing research that explores and addresses the effect of social determinants of health, particularly on marginalized communities, such as PSMI, and their priorities for change. Graduate students would benefit from specialized training in PAR’s use and application to a broad range of populations and diverse contexts, including research for policy change. Such efforts can create more space and opportunities for funding and training in participatory and transformative research approaches that address marginalized communities’ needs.

We also discussed five strategies in the articles reviewed to empower PSMI by engaging in PAR. These included building capacity, balancing power distribution, creating a collaborative environment, promoting peer support, and increasing engagement in the research process (Chen & Krupa, 2018). These strategies are consistent with the basic principles in the PAR literature to build the capacity of co-researchers and share power with them to address their experiences of injustice. Understanding barriers that prevent participation, using techniques to support participation, communicating clearly and consistently, and providing incentives and support are ways to enhance PSMI engagement in the research process. These are consistent with good practice guidelines for service user engagement, as Wallcraft and colleagues (2009) described. They stressed the need to involve PSMI from the outset of the project so that they have maximum influence and involvement. PAR researchers have cautioned that “inadequate time for involvement by peer researchers can render their participation superficial” (Lapadat et al., 2020, p. 7) and recommended that researchers include time for flexibility and delays in the research process (Lincoln et al., 2015).

## Limitations and Conclusion

The implications of our findings need to be seen in light of certain limitations. First, this review summarized and discussed information in the articles but did not include details (e.g., the extent of power-sharing between academic and community partners) because of space constraints. As our eligibility criteria included research published in English, we may have excluded other relevant literature. Lastly, within the premise of a scoping review, we limited our efforts to collate, summarize, and report results. We did not delve deeper into a critical analysis of the articles included.

In conclusion, this study contributed to the PAR literature by summarizing the nature and scope of PAR with PSMI. It highlighted five broad strategies to empower them, ways to enhance their meaningful participation in the research process, and recommendations to accommodate their needs better. Building on the work of Wallcraft and colleagues (2009), we recommend nurturing relationships among participants, building their capacity to make decisions concerning the research and action processes, finding ways to increase access to resources, accommodating their unique needs, and addressing barriers to participation. Finally, the PAR literature would benefit from reflexive accounts of the research process, reviews that explore changes attributable to PAR, and its utility in addressing specific research-related concerns, such as ethical considerations unique to engaging PSMI co-researchers and particular ways to accommodate their needs better in the research process.
